# Combining word embeddings to extract chemical and drug entities in biomedical literature

**DOI:** 10.1186/s12859-021-04188-3

**Published:** 2021-12-17

**Authors:** Pilar López-Úbeda, Manuel Carlos Díaz-Galiano, L. Alfonso Ureña-López, M. Teresa Martín-Valdivia

**Affiliations:** Department of Computer Science, Advanced Studies Center in Information and Communication Technologies (CEATIC), Universidad de Jaén, Campus Las Lagunillas s/n, 23071 Jaén, Spain

**Keywords:** Natural language processing, Named entity recognition, Concept indexing, Neural network, Word embeddings, SNOMED-CT

## Abstract

**Background:**

Natural language processing (NLP) and text mining technologies for the extraction and indexing of chemical and drug entities are key to improving the access and integration of information from unstructured data such as biomedical literature.

**Methods:**

In this paper we evaluate two important tasks in NLP: the named entity recognition (NER) and Entity indexing using the SNOMED-CT terminology. For this purpose, we propose a combination of word embeddings in order to improve the results obtained in the PharmaCoNER challenge.

**Results:**

For the NER task we present a neural network composed of BiLSTM with a CRF sequential layer where different word embeddings are combined as an input to the architecture. A hybrid method combining supervised and unsupervised models is used for the concept indexing task. In the supervised model, we use the training set to find previously trained concepts, and the unsupervised model is based on a 6-step architecture. This architecture uses a dictionary of synonyms and the Levenshtein distance to assign the correct SNOMED-CT code.

**Conclusion:**

On the one hand, the combination of word embeddings helps to improve the recognition of chemicals and drugs in the biomedical literature. We achieved results of 91.41% for precision, 90.14% for recall, and 90.77% for F1-score using micro-averaging. On the other hand, our indexing system achieves a 92.67% F1-score, 92.44% for recall, and 92.91% for precision. With these results in a final ranking, we would be in the first position.

## Background

Two traditional processes have been applied extensively in biomedical text mining. The first one is to extract those important and representative concepts within a specific domain. This task is commonly known as Named Entity Recognition (NER). The second process attempts to automatically assign an identifier to each previously extracted concept [1]. The cost of manual coding becomes expensive in cases where a more comprehensive or complete coding is required. In addition, this requires the expert to know the complete terminology in order to assign the correct code.

Natural Language Processing (NLP) can be a solution that gives fast, accurate and automated concept detection and coding that can provide important advances for the NER scientific community [2].

Chemical and drug named entity recognition is a fundamental step for further biomedical text mining and has received much attention recently. This task aims to automatically detect chemical and drug mentions in biomedical literature and is a great challenge to the scientific community for several reasons: there are several ways to refer to the same chemical or drug, abbreviations and acronyms are commonly used, symbols are often included in scientific publications and new chemicals and drugs are constantly and rapidly reported [3].

To support the coding of chemical and drug entities there are dictionaries, terminologies, and medical ontologies that allow this process to be carried out. SNOMED-CT is a reference terminology in the biomedical domain that allows a unique identifier code to be assigned to each recognized entity. Using this terminology in chemical and drug mentions we can create and maintain semantic interoperability of this clinical information [4].

In this study, we present the continuation of our participation in the Pharmacological Substances, Compounds and proteins and Named Entity Recognition (PharmaCoNER) challenge [5]. Our previous participation [6] did not obtain the expected results so we continue working to improve our systems. Since we already have the gold test we also present an in-depth error analysis which we carried out. This challenge proposes two sub-tasks for interested participants:

- NER offset and entity classification. The first evaluation scenario consists of the classical entity-based evaluation that requires the system outputs matching exactly the beginning and end locations of each entity tag, as well as matching the entity annotation type.
- Concept indexing. The second evaluation scenario consists of an entity linking or entity normalization task where for each entity the list of unique SNOMED-CT concept identifiers has to be generated. This is then compared to the manually annotated concept IDs corresponding to chemical compounds and pharmacological substances.

In order to carry out our NER task, we propose an approach based on neural networks using a combination of word embeddings. Our proposal is based on Recurrent Neural Networks (RNNs) or, more precisely, the bidirectional variant of Long Short Term Memory along with a stacked Conditional Random Fields decoding layer (BiLSTM-CRF) [7]. This architecture is chosen because it facilitates the processing of arbitrary length input sequences and enables the learning of long-distance dependencies, which is useful in the case of the NER task [8, 9]. Furthermore, our method proposes the combination of different types of word embeddings by concatenating each embedding vector to form the final word vectors. In this way, the probability of recognizing a specific entity in a text should be increased as different types of representation of that word are combined.

Our second approach is developed in order to assign a unique SNOMED-CT code to each entity. For this purpose, we have generated a hybrid method that mixes supervised and unsupervised approaches.

Our main contributions in this study can be summarized as follow:

- Combination of word embeddings for the integration of knowledge from different sources.
- Training of word embedding related to the biomedical domain in Spanish.
- Use of contextual string embeddings that model words as sequences of characters, contextualizing a word by the surrounding text.
- The application of a hybrid algorithm (supervised and unsupervised) in order to improve the concept indexing task.

The rest of the paper is structured as follows: in “Related work” section some previous related studies are described. The data we used to evaluate our experiments is described in “Data” section. The experimental methodology is laid out in “Methods” section. The evaluation of the results is presented in “Results and discussion” section. Finally, the analysis of errors is conducted in “Error analysis” section and conclusions are presented in “Conclusion” section.

## Related work

In the medical domain, NER systems identify clinical entities from narrative patient reports to support clinical and translational research. Various NER modules have been developed in general clinical NLP systems (e.g., MedLEE [10], MetaMap [11] and cTAKES [12]). Most of the existing clinical NLP packages are rule-based systems that rely on comprehensive medical vocabularies.

Drug and chemical name recognition, which seeks to recognize these types of mentions in unstructured medical texts and classify them into pre-defined categories, is a fundamental task of medical information extraction and medical relation extraction systems [13–15], and is the key to linking entities with terminologies available in the biomedical domain such as SNOMED-CT [16–19].

Recently, the clinical NLP community organized a series of open challenges with the focus on identifying chemical and drug entities from narrative clinical notes, including the Chemical compound and drug name recognition task (CHEMDNER) [20], the extraction of drug-drug interactions from biomedical texts task (DDIExtraction) [21] and the challenge Pharmacological Substances, Compounds and proteins Named Entity Recognition (PharmaCoNER) [5] presented at BioNLP 2019. These workshops are very useful because the participants use innovative and updated systems, offering a state-of-the-art approach to the tasks.

Approaches for NER can be classified into different categories [3]: dictionary-based, rule-based, and machine learning-based. Dictionary-based approaches identify drug names by matching drug dictionaries against given texts [22, 23]. For this purpose, it is necessary to start from a resource related to chemicals and drugs such as DrugBank [24], ChEBI [25] and PharmGKB [26], among others. Rule-based approaches use rules that describe the composition patterns or context of drug names [14, 27]. Finally, machine learning-based approaches usually formalize NER as a classification problem or a sequence-labeling problem. Each token is presented as features and is labeled by machine learning algorithms with a predefined category.

In the previous studies, the state-of-the-art chemical and drug entity recognition methods based on CRF have depended on effective feature engineering, i.e. the design of effective features using various NLP tools and knowledge resources [28–30]. Recently, deep learning has become prevalent in the machine learning research community in order to improve biomedical named entity recognition [31, 32]. Among others, the model of BiLSTM-CRF exhibits promising results [7, 33, 34]. These networks usually rely on word embeddings, which represent words as vectors of real numbers [35]. There are different types of word embeddings: classical [36, 37], character-level [38] and contextualized [39] which are commonly pre-trained over very large corpora to capture latent syntactic and semantic similarities between words.

Following the neural network proposed by Huang et al. [7], our work uses the BiLSTM-CRF network to detect chemicals and drugs in Spanish biomedical literature. We also evaluate the usefulness of each word embedding in two different ways: independently and in combination. Subsequently, we use a hybrid approach (supervised and unsupervised) to automatically assign a SNOMED-CT code to each entity detected.

## Data

**Fig. 1:**
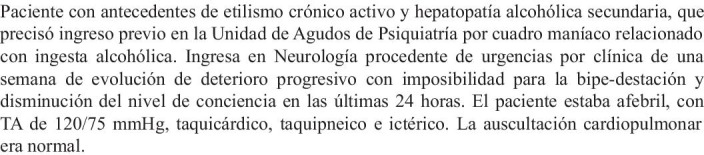
Sample fragment from the SPACCC corpus (see English translation in “Appendix A” Figure 6)

The dataset is named the Spanish Clinical Case Corpus [40] (SPACCC). The SPACCC corpus was created by collecting 1,000 clinical cases from SciELO [41] (Scientific Electronic Library Online), an electronic library that gathers electronic publications of complete full-text articles from scientific journals from Latin America, South Africa, and Spain. This type of narrative shows properties of both the biomedical and medical literature, as well as clinical records. Clinical cases cover a variety of medical disciplines such as oncology, cardiology, urology, infectious diseases, and pneumology, and these medical disciplines cover a diverse set of chemicals and drugs [5]. Figure 1 shows an example fragment of the SPACCC corpus.

Moreover, Table 1 shows some statistics about the corpus. As we can see the corpus is composed of a set of training (train), development (dev), and testing (test).

**Table 1:** Basic analysis of SPACCC corpus documents

	Train	Dev	Test
Number of documents	500	250	250
Avg sentences	25.14	25.85	25.69
No. tokens	202,901	96,869	100,963
No. unique tokens	18,623	12,170	12,442

The annotation of the entire set of entity mentions was carried out by medicinal chemistry experts and it includes the following four entity types or categories:

- NORMALIZABLES: mentions of chemical compounds and drugs that can be normalized or standardized in a unique identifier in the SNOMED-CT knowledgebase (e.g.: glucose, cholesterol, and creatinine).
- NO_NORMALIZABLES: mentions of chemical compounds and drugs that cannot be standardized (e.g.: pyrazolones, fluoroquinolones, and acid).
- PROTEINAS: peptides, proteins, genes, peptide hormones and antibodies (e.g.: transaminases, proteinuria, C3, and C4).
- UNCLEAR: pharmaceutical formulations, general treatments, chemotherapy programs, vaccines and a predefined set of general substances (e.g.: silymarin, melanin, alcohol, and tobacco). Mentions of this class will not be part of the entities evaluated by this challenge.

The dataset has an annotation guide [42] generated with the collaboration of practicing physicians and medicinal chemistry. In this guide, we can find all the information related to the annotation process in order to perform a more granular experiment. The statistics of the number of labels for each dataset are shown in Table 2.

**Table 2:** Distribution of labels in the SPACCC dataset

	Train	Dev	Test
NORMALIZABLES	2304	1121	973
NO_NORMALIZABLES	24	16	10
PROTEINAS	1405	745	859
UNCLEAR	89	44	34

## Methods

The workflow to address the proposed task in PharmaCoNER consists of two sequential steps, first detecting drug and chemical entities in Spanish clinical documents, and subsequently, the extracted entities must be assigned to a unique identifier code using SNOMED-CT terminology. In this section, we will evaluate the methods and resources used to carry out this task.

### Word embeddings

Word embeddings are a type of word representation that allows words with similar meanings to have a similar representation. The word representation of a document is an essential element in deep learning.

Specifically, word embedding is a technique in which individual words are represented as numerical vectors in a predefined vector space. Each word is mapped to one vector and embeddings are learned with neural networks, so this technique is often applied in the field of deep learning [43].

Different word embeddings have been combined to form the input layer to the proposed deep neural network. Each word representation used is explained in detail below:

#### Classic word embeddings

Classic word embeddings are static and word-level, meaning that each distinct word receives exactly one pre-computed embedding. Our experiments use FastText [44] embeddings trained over Spanish Wikipedia and size 100.

#### Training medical embeddings

There are biomedical word embeddings available for Spanish [45–47], however, they are not always available to the scientific community or obtain poor results due to the peculiarities of the language in a domain in which they were trained. Therefore, we generated new ones from existing corpora related to the biomedical domain in Spanish.

For this purpose, firstly we extracted the Spanish corpus from MeSpEN [48]. In addition, extra data in Spanish from different clinical information websites such as Mayo Clinic [49], the World Health Organization [50] and WebMD [51] was added to the former corpus. Finally, FastText was used to perform the training by applying the following setup: skip-gram model, 0.05 for the learning rate, size of 300 for the word vectors, 10 for the number of epochs, and 5 for the minimal number of word occurrences. This kind of embedding is available to the scientific community [52].

#### Contextual word embeddings

Contextualized word embeddings [53] capture latent syntactic-semantic information that goes beyond standard word embeddings. This representation treats text as distributions over characters and is capable of generating embeddings for any string of characters within any textual context, in other words, the same word will have different embeddings depending on its contextual use. For our experiments, we used the *pooled contextualized embeddings* proposed by Akbik et al. [54] to help with the recognition of chemicals and drugs. Pooled embeddings were originally trained on Spanish Wikipedia [55] by combining characters to form words and obtaining embeddings for them.

### Chemical components and drugs recognition

In order to extract the mentions of drugs and chemicals, we use the BiLSTM-CRF sequence labeling module proposed by Huang et al. [7]. Specifically, we used a BiLSTM with a sequential CRF layer.

Each type of embedding studied above is generated with a different method, which means that each word will be represented by aspects of knowledge based on the training corpus, and combining them could potentially improve performance.

**Fig. 2:**
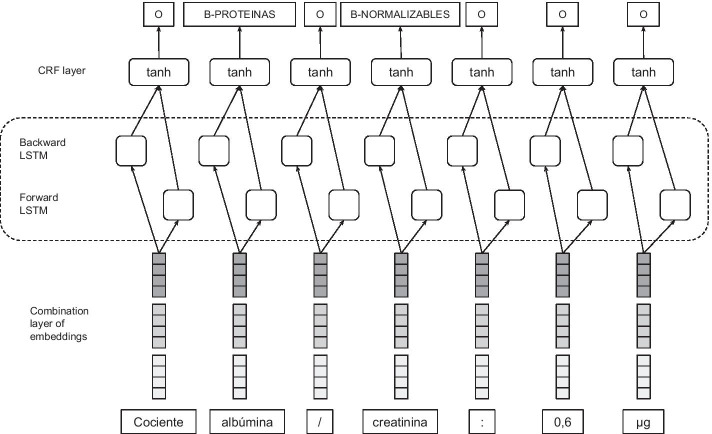
Proposed BiLSTM-CRF neural network using a combination of different word embeddings as an input layer. English translation: albumin/creatinine ratio: 0.6 μg

Given a sentence, the model predicts a label corresponding to each of the input tokens in the sentence. Firstly, through the embedding layer, the sentence is represented as a sequence of vectors *X*=($x_1$,$x_2$,...,$x_n$) where n is the length of the sentence. The combination of embeddings is the input to a BiLSTM layer. A forward LSTM computes a representation of the sequence from left to right at every word, and another backward LSTM computes a representation of the same sequence in reverse. Then a *tanh* layer is used to predict confidence scores for the word, having each of the possible labels as the output scores of the network. Finally, instead of modeling tagging decisions independently, the CRF layer is added in order to decode the best tag of all the possible tags. Figure 2 shows the proposed architecture based on a BiLSTM-CRF.

For the implementation, we employed Flair [56]. Flair is a simple framework for NLP tasks including NER which provides the BiLSTM-CRF architecture. The neural network is used with the following configuration: learning rate as 0.1, dropout as 0.5, maximum epoch as 150, 300 neurons with *tanh* activation function, and a batch size of 32.

For the entity recognition task, the annotations provided were encoded by using the BIO tagging scheme. Thus each token in a sentence was labeled with B (beginning token of an entity), I (inside token of an entity), or O (non-entity). This scheme is the most popular in the NER task.

### Concept indexing with SNOMED-CT

According to the second task proposed by PharmaCoNER, a unique SNOMED-CT code has to be assigned to each previously extracted entity. For this task, we use a hybrid system that combines supervised and unsupervised methods. On the one hand, the supervised process makes use of the terms included in the training set. This process is limited to training concepts and would ignore those that are new, and for that reason, it is necessary to add the unsupervised process to cover those concepts not seen before. Specifically, this supervised method is a dictionary-based approach with SNOMED-CT concepts included in the training.

**Fig. 3:**
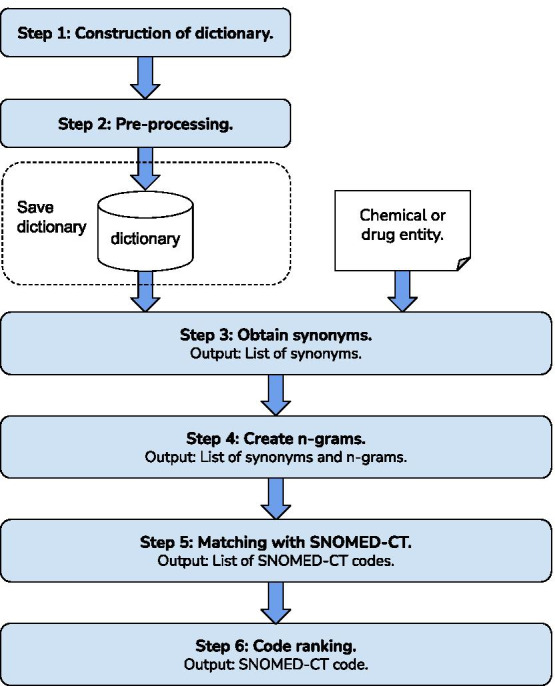
Workflow for assigning a SNOMED-CT code to an entity

On the other hand, for the unsupervised process we continue to explore the architecture created for our previous study [6] based on the six steps shown in Fig. 3. This architecture starts from the pre-processing and ends with the assignment of a SNOMED-CT code using Levenshtein distance. The steps followed until the SNOMED-CT identifier is obtained are detailed below:

1. *Construction of a dictionary* The first step of this workflow is the construction of a dictionary. The goal of this dictionary is to create a list of synonyms to help obtain a code of the terminology. This dictionary was created using different sources of information related to chemicals and drugs including Wikidata, the Spanish Medical Abbreviation DataBase [57] (AbreMES-DB), Nomenclator for prescription drugs [58], Chemical symbols in Spanish and products and substances in Spanish SNOMED-CT. All these sources of information have something in common, they all contain synonyms, acronyms, or other ways of referring to the same entity.
2. *Pre-processing* For the synonym and the entity to match correctly, they should be standardized in the same way. The pre-processing carried out for both the dictionary and the recognized entity is the following: change the text to lowercase, remove accents, lemmatize, remove punctuation marks and remove stop-words.
3. *Obtain synonyms* At this point the recognized entity is matched with all the dictionary entries. In the case that we can match them, we will increase the list of possible synonyms in order to have more options to find the concept in SNOMED-CT. For instance, the entity “GGT” contains several synonyms such as “*gama glutamil transferasa*” (gamma-glutamyl transferase), “GGTP”, “*gamma-glutamiltransferasa*” (gamma-glutamyltransferase), and “*gamma GT*”. In this case, “GGT” is not a concept included in SNOMED-CT, however, we can find “*gamma-glutamiltransferasa*” with ID 60153001.
4. *Create n-grams* The chemicals and drugs extracted are often multi-word and do not match correctly. To avoid this situation we decided to create n-grams, where *n* is the size of the multi-word concept with all possible word combinations. The output of this step will be the combination of the list of new n-grams and the list of possible synonyms of the entity generated in the previous step. With this step, we can solve problems such as the following: the entity “*dímero D*” (D-dimer) is a protein that can also appear as “*D dímero*” and the entity “*proteína A amiloide*” (amyloid protein A) such as “*proteína amiloide A*”
5. *Matching with SNOMED-CT* Each concept on the previously generated list is matched with each SNOMED-CT using the library named Hunspell [59]. This library contains a function that provides a list of suggested concepts.
6. *Code ranking* Since we have to return a single SNOMED-CT ID, the list of suggested concepts from the previous step must be ranked. For this purpose, we use the Levenshtein distance. Finally, we chose the SNOMED-CT concept that has the least distance from the input text.

## Results and discussion

This section presents the results obtained using the methodologies proposed previously. For both scenarios (NER and concept indexing), the primary evaluation metrics used consisted of standard measures from the NLP community, namely micro-averaged precision, recall, and balanced F1-score. To compute the metrics we used the evaluation library proposed by the organizers of the PharmaCoNER challenge [60] where TP (True Positive) is the set of samples that have exactly matched the start and end locations of each entity label, as well as the type of entity annotation with the gold standard, FP (False Positive) refers to a system response that does not exist in the gold annotation, and FN (False Negative) is a golden annotation that is not captured by a system.

**Table 3:** Micro-averaged performance for chemical and drug recognition task using BiLSTM-CRF approach

	Precision (%)	Recall (%)	F1-score (%)
Based on BERT (Xiong et al. [61])	91.23	90.88	91.05
Classic WE + Contextual WE + Medical WE	91.41	90.14	90.77
Medical WE	87.94	86.24	87.08
Contextual WE	88.74	85.22	86.95
Classic WE	86.53	83.46	84.96
CRF + features (López-Úbeda et al. [6])	88.51	69.81	78.06

According to the first scenario proposed in PharmaCoNER the systems address the NER task, wherein the entities proposed by the organizers are three: NORMALIZABLES, NO_NORMALIZABLES, and PROTEINAS. Table 3 shows the performances of the BiLSTM-CRF based NER system on the SPACCC corpus using different word embeddings representations. As we can see, the first row describes the best result obtained in the PharmaCoNER challenge. This system developed by Xiong et al. [61] uses a BERT-based system. The last row of the table presents our best results sent to the PharmaCoNER challenge, and this system was CRF-based and has features named “*Run 2: CRF + basic features + features base on medical terminology*” [6]. To judge the statistical significance of the differences between the performance of *Classic WE + Contextual WE + Medical WE* system and *CRF + features* [6] system, we performed McNemar’s test. The test offered a p<0.05 suggesting that our new model provides statistically significant results recognizing pharmacological entities.

We first carry out an experiment using each of the word embeddings explained in “Word embeddings” individually: classic word embeddings (WE), contextual WE, and trained medical WE. As we can see, the use of each of them already improves our previous result. In terms of recall using classic WE we achieved a 13.65% increase over the result with *CRF + features*, 15.41% using contextual WE, and 16.43% with medical WE. This is the key to improving the F1-score as the precision obtained differs little. Usually, a large leap in recall leads to a decrease in precision, but according to our results, there was only a small drop in precision when going from the CRF system to the classic WE system and medical WE (BiLSTM-CRF), however, this drop in precision disappeared when using contextual WE.

Subsequently, we propose a combination of word embeddings to represent the words of the corpus. Our best system proposes combining the three types of embeddings seen above separately and together they achieve 90.77% of F1-score, 91.41% of precision, and 90.14% of recall. This system is close to achieving the best results of the challenge. In terms of precision we obtain a 0.18% improvement over the best system, in contrast, we obtain 0.44% less in recall and 0.28% less in F1-score.

**Fig. 4:**
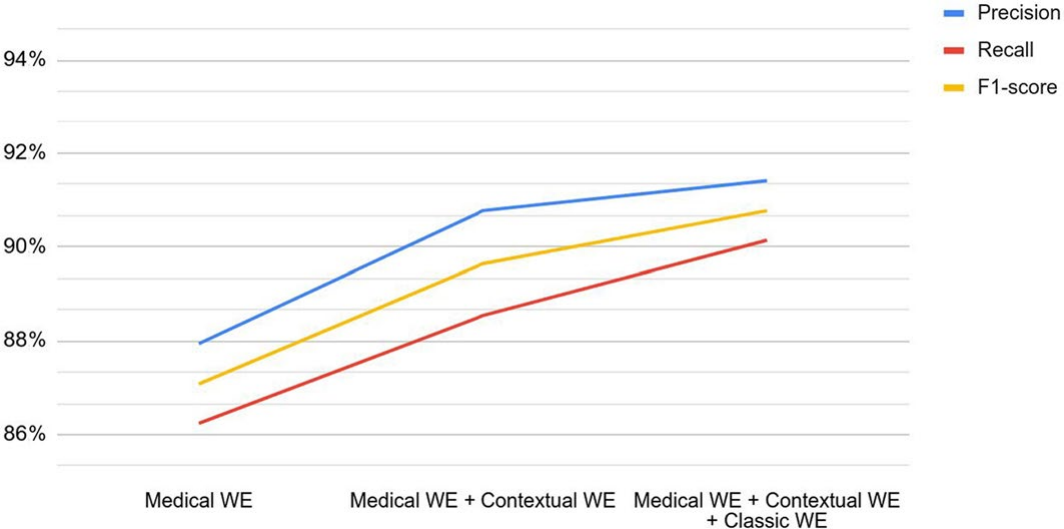
Increased results by concatenating word embeddings for NER task

The combination of word embeddings adds relevant information in order to represent each word, and the neural network is able to recognize chemicals and drugs efficiently. Figure 4 shows the improvement regarding precision, recall, and F1-score by adding new word embeddings. The graph shows in the first iteration the use of medical word embeddings, then we concatenate medical WE and contextual WE and finally show the results of all three types together.

In terms of time consumed, we found that combining three types of word embeddings requires 5 hours of processing on a single Tesla-V100 32 GB GPU with 192 GB of RAM. However, using one word embedding the performance requires approximately 2 hours.

Regarding the second task proposed by the PharmaCoNER challenge, the results are shown in Table 4. This task consists of assigning a SNOMED-CT identifier to each entity recognized in the previous task. It is important to emphasize that we always use the same system (see “Concept indexing with SNOMED-CT” section) to assign the unique identifier, i.e., we use the annotated entities in the previous task to assign a SNOMED-CT code.

**Table 4:** Micro-averaged performance for the concept indexing task

	Precision (%)	Recall (%)	F1-score (%)
Classic WE + Contextual WE + Medical WE	92.91	92.44	92.67
Rule + Dictionary-based method (León et al. [62])	91.11	92.08	91.59
Contextual WE	91.11	91.93	91.34
Medical WE	92.16	90.15	91.17
Classic WE	92.13	89.34	90.14
CRF + features [6]	82.89	61.84	70.83

As we can see in Table 4, the best result obtained was also using the combination of word representations. With this system, we achieved 92.67% of F1-score, 92.91% of precision, and 92.44% of recall. The system we sent before the challenge obtained 70.83% of F1-score so we improved this result by 21.84%. This result shows a substantial improvement which is due to several reasons: a new supervised method has been developed for the generation of the hybrid approach, and the task of NER has been enhanced. With our new method, we would reach the first position in the PharmaCoNER concept indexing task, surpassing León and Ledesma [62] by 1.08% of F1-score. Compared to the previous system submitted to the PharmaCoNER challenge [6], with the new system, we obtain improvements in both precision and recall.

### Error analysis

The main purpose of this section is to carry out an error analysis in order to identify the weaknesses of our system. For this purpose, we conducted two different studies: the first one to obtain the TP, FP, and FN in the NER task, and the second one to present some examples of misclassification and principal findings in both cases (NER task and concept indexing).

This error analysis has been carried out by analyzing the errors and successes produced by our best system method (Classic WE + Contextual WE + Medical WE) recognizing mentions of chemicals and drugs in the medical science literature.

A fine-grained evaluation of the systems can be defined in terms of comparing the response of the system against the golden annotation [63]. The evaluation of our system considering these different categories of errors is shown in Table 5. As we can notice, the system learns well the entities annotated as NORMALIZABLES since it correctly annotates 893 (TP) of 973, which means that it fails in 80 (FN). On the other hand, our system labels 58 entities with this category when in fact the annotation is not correct. The same situation occurs with the category PROTEINAS, the system fails in 91 entities (FN). However, due to the few examples of NO_NORMALIZABLES our method only labels 3 (TP) of 10 entities. The results of the table suggest that the system is more accurate in identifying entities the more mentions the training corpus contains.

**Table 5:** Fine-grained evaluation considering different errors categories in the NER task

	Total	TP	FP	FN
NORMALIZABLES	973	893	58	80
NO_NORMALIZABLES	10	3	1	7
PROTEINAS	859	768	98	91

**Fig. 5:**
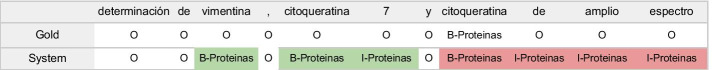
Example of FN in the PharmaCoNER corpus comparing the gold output and the output of our system. English translation: determination of vimentin, cytokeratin 7 and broad-spectrum cytokera

Regarding some errors produced by our system, we wanted to show some examples of fragments of the SPACCC corpus in which our system misclassified. In Fig. 5 we show an FN since our system identifies the entity “*citoqueratina de amplio espectro*” (broad-spectrum cytokeratin) as PROTEINAS but in the gold system the correct entity is “*citoqueratina*” (cytokeratin). This is a clear example of how our system, although it is correct with the label (PROTEINAS), does not match well the beginning and the end of the entity. Errors such as the latter shown (no matching start or end of the entity but the matching type of entity) occur about 81 times. Another error of this type is found with the entity annotated on the gold as “*antigangliósidos GM1 y GD1b*” (GM1 and GD1b antigangliosides) where our system recognizes “*antigangliósidos GM1*” and “*D1b*” independently. This means that the system produces three error types: one FN and two FP, because the originating entity has not been annotated by the system (one FN) and our system has produced two entities that are not in the standard gold (two FP). We could treat entities that are marked as consecutive but are independently identified by our system or the opposite, for instance, our system recognizes “*isoenzimas*” (isoenzymes) and “*FA*” but the correct entity is “*isoenzimas de FA*”.

On the other hand, in order to better understand the entities mislabeled by the neural network we performed a manual inspection on a subset of the data and recorded some of the results in Table 6. This table shows the true label, the category predicted by the neural network, and some examples of misclassified entities. As we can see, the proposed method does not usually label the category NO_NORMALIZABLES since there are few examples of training.

**Table 6:** Examples of misclassified entities in the NER task

True label	Predicted label	Entities
NORMALIZABLES	PROTEINAS	*Actocortina*, *ADR* (RDA), TG
	O	*Carbohidratos* (carbohydrates), BH4, *tiacídicos* (thiazides), *calcio* (calcium), CTX
NO_NORMALIZABLES	NORMALIZABLES	Ora-Sweet, harvoni, endoperox
	O	Ora-Plus, McGhan
PROTEINAS	NORMALIZABLES	*Progesterona* (progesterone), *hormonas* (hormones), *vasopresina* (vasopressin)
	O	A.S.T, DHL, CLL-K
O	NORMALIZABLES	*Azúcar* (sugar), *cimetidina* (cimetidine), *anión* (anion), *loprofín* (loprofin)
	NO_NORMALIZABLES	Aproten
	PROTEINAS	*PCE* (ECP), *protínograma* (prothinogram), *CHCM* (MCHC), LDH

Regarding the task of indexing concepts using SNOMED-CT terminology, we manually selected some error cases. Table 7 shows some examples of entities that our system was not able to annotate. As we can see in these examples, they are acronyms that were not included in the synonym dictionary. Moreover, the description of the concepts in SNOMED-CT often varies from the annotated entity, which is difficult to find using the Levenshtein distance.

**Table 7:** Examples of entities incorrectly indexed by the unsupervised machine learning method

Entity	SNOMED-CT code	SNOMED-CT description
cd 31	4167003	*Antígeno linfocitario CD31* (lymphocyte antigen CD31)
*Proteínas totales* (total proteins)	395835001	*Proteína plasmática* (plasma protein )
*Anti-MBG* (anti-GBM)	11353004	*Anticuerpo antimembrana basal glomerular* (anti glomerular basement membrane antibody)

There are entities that the method has not been able to index correctly such as the *beta-HCG* entity. To this entity, the system assigned the pair (412126005, *gonadotrofina corionica humana*/human chorionic gonadotropin), but according to the gold standard test the correct pair is (40940006, *gonadotrofina corionica humana subunidad beta*/human chorionic gonadotrophin beta subunit). Another case of error occurred with the *antiRNA* entity, the system marked the pair (47646004, *antiarina*) but the correct one is (444236000 *anticuerpo anti-ácido ribonucleico*/anti-ribonucleic acid antibody) .

Finally, we would also like to highlight some difficult cases in which the unsupervised machine learning system has been able to annotate correctly. Table 8 shows some of these cases. Note, for example, that the entity detection system recognizes “*adriamicina*” (adriamycin) as an entity but in the SNOMED-CT description it appears as “*doxorrubicina*” (doxorubicin). We consider that this matching would be hard to detect if we did not have a list of synonyms previously created for that word.

**Table 8:** Examples of entities correctly indexed by the unsupervised machine learning method

Entity	SNOMED-CT code	SNOMED-CT description
*Adriamicina* (adriamycin)	372817009	*Doxorrubicina* (doxorubicin)
EMA	103092003	*Antígeno cancerígeno* (carcinogenic antigen) 15 3
AA	40185008	*Proteína amiloide sérica A* (serum amyloid protein A)

## Conclusion

As we proposed in our previous paper [6], we continue to study sophisticated neural networks with the use of word embeddings. Word embeddings are word representations that allow us to capture the context of a word in a sentence by providing relevant information. In this paper, we present the combination of them in order to improve the NER system.

Our proposal method follows a deep learning-based approach for NER in Spanish health documents. It is focused on the use of a BiLSTM-CRF neural network where different word embeddings are combined as an input to the architecture. Then this neural network is trained by using the annotated datasets provided by the organizers of the PharmaCoNER challenge.

Our main goal was to prove the performance of different types of word embeddings for the NER task: classic word embeddings trained with fastText on the Spanish Wikipedia corpus, contextual embeddings that provide extra information about the context, and other word embeddings trained by ourselves adding more sources of information related to the biomedical domain. With the concatenation of these word embeddings, we achieved results of 91.41% for precision, 90.14% for recall, and 90.77% for F1-score which is an improvement of 12.71% in the F1-score concerning our previous paper. Our NER method exceeds by 0.18% the precision of the best team at PharmaCoNER. With the results obtained, we would be close to the first positions in a final classification.

Concerning the task of concept indexing, we propose a hybrid method based on supervised and unsupervised machine learning. On the one hand, the supervised approach uses the training set to learn SNOMED-CT codes, on the other hand, the unsupervised approach consisted of a 6-step methodology. In this methodology, a synonym dictionary is generated to improve indexing, especially in the case of acronyms such as TSH (liothyronine) or CEA (carcinoembryonic antigen). Our indexing system achieved a 92.67% F1-score, 92.44% recall, and 92.91% precision. The results in this task are promising since we surpassed the best team presented at PharmaCoNER.

For future work, we plan to improve our entity detection system using new transfer learning techniques. In addition, there are available pre-trained models for the biomedical domain such as BioBERT that could be taken into consideration. Although BioBERT is in English, an ideal scenario would be the generation of a new model for Spanish. Regarding concept indexing, we plan to process SNOMED-CT in Spanish more thoroughly, for example using all SNOMED-CT concepts not only the semantic types products and substances, checking the validation of the concept in the last version if there have been changes in the description in the last version, and so on.

## Figures and Tables

**Fig. 6:**
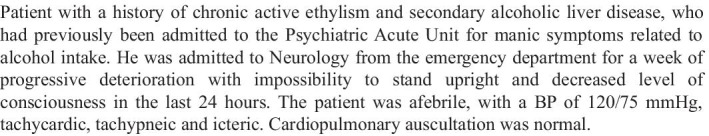
English sample fragment from the SPACCC corpus

## Data Availability

The datasets analyzed during the current study are available in the PharmaCoNER repository at https://temu.bsc.es/pharmaconer/index.php/datasets/.

## Appendix A: Translation cases

See Fig. 6.

## Abbreviations

NLP: Natural language processing
NER: Named entity recognition
SNOMED-CT: Systematized nomenclature of medicine-clinical terms
RNN: Recurrent neural network
BiLSTM: Bidirectional long short-term memory
CRF: Conditional random field
SPACCC: Spanish Clinical Case Corpus
TP: True positive
FP: False positive
FN: False negative
WE: Word embedding
